# An Intelligent Model for Pairs Trading Using Genetic Algorithms

**DOI:** 10.1155/2015/939606

**Published:** 2015-08-03

**Authors:** Chien-Feng Huang, Chi-Jen Hsu, Chi-Chung Chen, Bao Rong Chang, Chen-An Li

**Affiliations:** ^1^Department of Computer Science and Information Engineering, National University of Kaohsiung, Kaohsiung 811, Taiwan; ^2^Department of Electrical Engineering, National Chiayi University, Chiayi City 60004, Taiwan

## Abstract

Pairs trading is an important and challenging research area in computational finance, in which pairs of stocks are bought and sold in pair combinations for arbitrage opportunities. Traditional methods that solve this set of problems mostly rely on statistical methods such as regression. In contrast to the statistical approaches, recent advances in computational intelligence (CI) are leading to promising opportunities for solving problems in the financial applications more effectively. In this paper, we present a novel methodology for pairs trading using genetic algorithms (GA). Our results showed that the GA-based models are able to significantly outperform the benchmark and our proposed method is capable of generating robust models to tackle the dynamic characteristics in the financial application studied. Based upon the promising results obtained, we expect this GA-based method to advance the research in computational intelligence for finance and provide an effective solution to pairs trading for investment in practice.

## 1. Introduction

In the past decades, due to the inefficacy of traditional statistical approaches, such as regression-based and factor analysis methods for solving difficult financial problems, the methodologies stemming from computational intelligence, including fuzzy theory, artificial neural networks (ANN), support vector machines (SVM), and evolutionary algorithms (EA), have been developed as more effective alternatives to solving the problems in the financial domain [1, 2].

Among the CI-based techniques studied for finance, the models may be classified as two major areas of applications: (1) stock selection, portfolio management, and optimization [3–6] and (2) prediction of financial time series [7, 8]. For the first category, earlier research works include the fuzzy multiple attribute decision analysis for portfolio construction [9]. Zargham and Sayeh [10] employed a fuzzy rule-based system to evaluate a set of stocks for the same task. Chapados and Bengio [11] trained neural networks for estimation and prediction of asset behavior to facilitate decision-making in asset allocation.

In EA applications along this line of research, Becker et al. [12] employed genetic programming (GP) to develop stock ranking models for the U.S. market. Lai et al. [13] used a double-stage GA to select stocks from the Shanghai stock exchange for the time period of years 2001 to 2004. In Lai et al.'s work, ROCE, EPS, PE, and liquidity ratios are used to rank stocks, and they used the GA to compute the optimal percentage of capital assigned to each of the assets. Lai et al. then concluded that their GA-based optimization method is more effective for financial applications than fuzzy or artificial neural networks. Recently, Huang [5] devised a hybrid machine learning-based model to identify promising sets of features and optimal model parameters; Huang's model was demonstrated to be more effective than the benchmark and some traditional statistical methods for stock selection. To improve the performance of the single-objective GA-based models, more recently, Chen et al. [14] proposed a multiobjective GA-based method for the goals of increasing investment return and reducing the risk simultaneously. In that approach, the authors used the nondominated sorting to search for nondominated solutions and showed that the multiobjective method outperformed the single-objective version proposed by Huang [5].

Another popular study of computational intelligence has been particularly concerning the prediction of financial time series. A certain amount of research employs network learning techniques, including feed-forward, radial basis function or recurrent NN [7], and SVM [8]. Other intelligent methods, such as genetically evolved regression models [15] and inductive fuzzy inference systems [16], were also available in the literature.

Pairs trading [17] is an important research area of computational finance that typically relies on time series data of stock price for investment, in which stocks are bought and sold in pairs for arbitrage opportunities. It is a well-known speculative strategy in the financial markets developed in the 1980s and has been employed as one important long/short equity investment tool by hedge funds and institutional investors [18]. Although there has been a significant amount of CI-based studies in financial applications, reported CI-based research for pairs trading is sparse and lacks serious analysis. To date, many existing works along this line of research rely on traditional statistical methods such as the cointegration approach [19], the Kalman filters [20, 21], and the principle component analysis [18]. In the CI area, Thomaidis et al. [17] employed a method of neural networks for the paired companies of Infosys and Wipro in India and accomplished reasonable return on investment using the pair of stocks. Saks and Maringer [22] used genetic programming for various pairs of stocks in Eurostoxx 50 equities and also found good pair-trading strategies.

Although there exist these previous CI-based studies for pairs trading, they lacked serious analysis such as the method of temporal validation used in [5, 23] for further evaluation of the robustness of the trading systems. In addition, in these previous studies, the trading models were constructed using only two stocks as a trading pair; here, we propose a generalized approach that uses more than two stocks as a trading group for arbitrage in order to further improve the performance of the models. In this study, we also employ the GA for the optimization problems in our proposed arbitrage models. In a past study [23], Huang et al. compared the traditional linear regression and the GA for the task of stock selection and showed that the GA-based model is capable of outperforming the linear regression model. Motivated by this research work, we thus intend to employ the GA to optimize our intelligent system for pairs trading, and the experimental results will show that our proposed GA-based methodology is promising in outperforming the benchmark. Furthermore, in contrast to traditional pairs-trading methods that aim at matching pairs of stocks with similar characteristics, we also show that our method is able to construct working trading models for stocks with different characteristics. In this study, we also investigate the robustness of our proposed method and the results show that our method is indeed effective in generating robust models for the dynamic environment of the pairs-trading problem.

This paper is organized into four sections.  Section 2 outlines the method proposed in our study. In Section 3, we describe the research data used in this study and present the experimental results and discussions.  Section 4 concludes this paper.

## 2. Materials and Methods

In this section, we provide the relevant background and descriptions for the design of our pairs-trading systems using the GA for model optimization.

### 2.1. Pairs Trading

Pairs trading is widely assumed to be the “ancestor” of statistical arbitrage, which is a trading strategy to gain profit from pricing discrepancies in a group of stocks [17]. Traditional decision-making for investment typically relies on fundamentals of companies to assess their value and price their stocks, accordingly. As the true values of the stocks are rarely known, pairs-trading techniques were developed in order to resolve this by investing stock pairs with similar characteristics (e.g., stocks from the same industry). This mutual mispricing between two stocks is theoretically formulated by the notion of spread, which is used to identify the relative positions when an inefficient market results in the mispricing of stocks [18, 21]. As a result, the trading model is usually market-neutral in the sense that it is uncorrelated with the market and may produce a low-volatility investment strategy.

A typical form of pairs trading of stocks operates by selling the stock with a relatively high price and buying the other with a relatively low price at the inception of the trading period, expecting that the higher one will decline while the lower one will rise in the future. The price gap of the two stocks, also known as spread, thus acts as a signal to the open and close positions of the pairs of stocks. During the trading period, position is opened when the spread widens by a certain threshold, and thereafter the positions are closed when spread of the stocks reverts. The objective of this long-short strategy is to profit from the movement of the spread that is expected to revert to its long-term mean.

Consider initial capital *X*_0_, with an interest rate of *r* per annum and a frequency of compounding *n* in a year; the capital *X* after a year may be expressed as

(1) $$\begin{array}{c} X={\left(1+\frac{r}{n}\right)}^{n}\cdot X_{0}. \end{array}$$

If the frequency of compounding *n* gets arbitrarily large, we have

(2) $$\begin{array}{c} \underset{n\to \infty }{\lim}{\left(1+\frac{r}{n}\right)}^{n}=e^{r}. \end{array}$$

In the case of continuously compounded return, the process of capital growth is defined as

(3) $$\begin{array}{c} X=e^{r}\cdot X_{0}. \end{array}$$

Therefore, the continuously compounded rate *r* is calculated by taking the natural logarithm as follows:

(4) $$\begin{array}{c} \ln\left(\frac{X}{X_{0}}\right)=r, \end{array}$$

where ln⁡(·) is the natural log function.

Now consider the two price time series, *P*_1_(*t*) and *P*_2_(*t*), of two stocks *S*_1_ and *S*_2_ with similar characteristics, the process of a pairs-trading model can be described as follows [18]:

(5) $$\begin{array}{c} \ln\left(\frac{P_{1}\left(t\right)}{P_{1}\left(t_{0}\right)}\right)=\alpha \left(t-t_{0}\right)+\beta \ln\left(\frac{P_{2}\left(t\right)}{P_{2}\left(t_{0}\right)}\right)+\gamma \left(t\right), \end{array}$$

where *γ*(*t*) is a stationary, mean-reverting process; the drift *α* is small compared to the fluctuations of *γ*(*t*) and can be neglected in many applications.

The rationale behind the mean-reverting process is that there exists a long-term equilibrium (mean) for the spread. The investor may bet on the reversion of the current spread to its historical mean by selling and buying an appropriate amount of the pair of the stocks. As (5) shows, one expects the returns of stocks *S*_1_ and *S*_2_ to track each other after controlling for proper  *β*. This model suggests an investment strategy in which one goes long 1 dollar of stock *S*_1_ and short *β* dollars of stock *S*_2_ if *γ*(*t*) is small. Conversely, if *γ*(*t*) is large, one takes an opposite strategy that goes short *S*_1_ and long *S*_2_. As a result, the return of the long-short portfolio may oscillate around a statistical equilibrium.

In real-world practice, the return of the long-short portfolio above for a period of time may be calculated as follows:

(6) $$\begin{array}{c} {\text{Ret}}_{t}=\ln\left(\frac{P_{1}\left(t\right)}{P_{1}\left(t-1\right)}\right)-\beta \ln\left(\frac{P_{2}\left(t\right)}{P_{2}\left(t-1\right)}\right), \end{array}$$

where *P*_1_(*t*) and *P*_1_(*t* − 1) denote the price of stock *S*_1_ where we are long at time *t* and *t* − 1, respectively; and *P*_2_(*t*) and *P*_2_(*t* − 1) denote the price of stock *S*_2_ where we are short at time *t* and *t* − 1, respectively.

The pairs-trading method can be generalized to a group of stocks in which mispricing may be identified through a proper combination of assets whose time series is* mean-reverting*. Consider a set of assets, *S*_1_, …, *S*_*m*_, and the corresponding time series of stock prices, *P*_1_(*t*),…, *P*_*m*_(*t*); a statistical mispricing may be considered as a linear combination *B* = (*β*_1_, *β*_2_,…, *β*_*m*_) such that

(7) $$\begin{array}{c} \ln\left(\frac{P\left(t\right)}{P\left(t_{0}\right)}\right)=\alpha \left(t-t_{0}\right)+\overset{m}{\underset{i=1}{\sum }}\beta_{i}\ln\left(\frac{P_{i}\left(t\right)}{P_{i}\left(t_{0}\right)}\right)+\gamma \left(t\right), \end{array}$$

where *γ*(*t*) is a mean-reverting process and vector *B* represents the proportions of one's capital assigned to each asset in the portfolio. Mean reversion in the equation above refers to the assumption that both the high and low prices of the synthetic asset *P* are temporary and that its price tends to move toward its average price over time.

### 2.2. Trading Systems

#### 2.2.1. Market Timing Models

In this work, the long-term mean of an asset's price in the mean-reverting process may be modeled by the celebrated moving average [24], which is the average price of an asset in a specified period. Let *P*(*t*) be the price of a stock at time *t*. The moving average at time *t*, the mean of the prices corresponding to the most recent *n* time periods, is defined as

(8) $$\begin{array}{c} {\text{MA}}_{n}\left(t\right)=\frac{1}{n}\overset{n}{\underset{i=1}{\sum }}P\left(t-i+1\right). \end{array}$$

In this study, we employ the Bollinger Bands [24] to determine if the spread of a pair of stocks departs from its dynamic average value. Typically, the Bollinger Bands prescribe two volatility bands placed above and below a moving average, in which volatility may be defined as a multiple of the standard deviation of the prices in the past. Formally the Bollinger Bands can be defined as follows:

(9) $$\begin{array}{c} {\text{MB}}_{n}\left(t\right)={\text{MA}}_{n}\left(t\right);\\ {\text{UB}}_{n}\left(t\right)={\text{MB}}_{n}\left(t\right)+k*\sigma_{n}\left(t\right);\\ {\text{LB}}_{n}\left(t\right)={\text{MB}}_{n}\left(t\right)-k*\sigma_{n}\left(t\right), \end{array}$$

where *σ*_*n*_(*t*) is the standard deviation of the prices, at time *t*, over the past *n* time periods; *k* ∈ *R* is a parameter used to control the width of the upper and lower bands to the moving average.

An important component of a successful trading system is to construct models for market timing that prescribe meaningful entry and exit points in the market. In this study, we will use the moving averages and Bollinger Bands to develop a trading system, which is described in the next subsection.

#### 2.2.2. Trading Strategy and Performance Evaluation

We calculate the spread for the synthetic asset generated by *m* stocks as

(10) $$\begin{array}{c} P\left(t\right)=\;\overset{m}{\underset{i=1}{\sum }}\beta_{i}P_{i}\left(t\right), \end{array}$$

where *P*_*i*_(*t*), *i* = 1,…, *m*, is the price of stock *i* at time *t*, and *β*_*i*_'s are the model parameters of generalized pairs trading to be estimated.

In this work, we designate the trading strategy for one to buy (sell) the spread right after it gets *x* standard deviations below (above) its mean value and the position is closed right after the spread gets closer than *y* standard deviations to its mean, where *x*, *y* ∈ *R* and *x* > *y* > 0.

Here we evaluate the performance of a trading system in terms of its compounded return, which is to be determined by the relevant parameters of the trading models employed. We first define the return of a trading system for the *l*th trade as *R*_*l*_(*θ*) ∈ *R*, where *θ* denotes the set of the model parameters. Then the performance metric we use here is through the total cumulative (compounded) return, *R*_*c*_, where *R*_*c*_ is defined by the product of the returns over *z* consecutive trades as

(11) $$\begin{array}{c} R_{c}=\overset{z}{\underset{l=1}{\prod }}R_{l}. \end{array}$$

Therefore, in the process of capital growth, the capital *X*_*z*_ at the end of *z* trades is

(12) $$\begin{array}{c} X_{z}=R_{c}X_{0}, \end{array}$$

where *X*_0_ represents the initial capital.

### 2.3. Optimization of Trading Systems

Given the market timing and pairs-trading models, the performance of a trading system shall be enhanced by suitable values of the corresponding model parameters. For the market timing models, the parameters include the period *n* for the moving average and parameters *x* and *y* for the Bollinger Bands that controls the multiples of the standard deviations of the moving average for entry and exit points. For the pairs-trading model, the parameters consist of the set of the weighting terms (*β*_*i*_'s) in the syntactic asset from (10). In this study, we propose using genetic algorithms (GA) for the search of optimal parameters of the trading system. We will describe the basics of GA as well as our proposed optimization scheme in the following.

Genetic algorithms [25] have been used as computational simulation models of natural evolutionary systems and as adaptive algorithms for solving complex optimization problems in the real world. The core of this class of algorithms lies in the production of new genetic structures, along the course of evolution, that provide innovations to solutions for the problem. Typically, the GA operate on an evolving population of artificial agents whose composition can be as simple as a binary string that encodes a solution to the problem at hand and a phenotype that represents the solution itself. In each iteration, a new generation is created by applying crossover and mutation to candidates selected as the parents. Evolution occurs by iterated stochastic variation of genotypes and selection of the fit phenotypes in an environment based on how well the individual solutions solve a problem.

**Figure 1:**
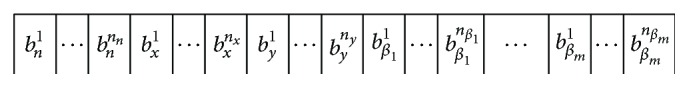
Chromosome encoding.

In our proposed encoding design, the composition of a chromosome is devised to consist of four portions that encode the period parameter *n* for the moving average, the multiples *x* and *y* of standard deviations for the Bollinger Bands, and the set of the weighting coefficients (*β*_*i*_'s) for the pairs-trading model from (10). Here we use the binary coding scheme to represent a chromosome in the GA. In Figure 1, loci *b*_*n*_^1^ through *b*_*n*_^*n*_*n*_^ represent the encoding for the period *n* of moving average. Loci *b*_*x*_^1^ through *b*_*x*_^*n*_*x*_^ and *b*_*y*_^1^ through *b*_*y*_^*n*_*y*_^ represent the encoding of *x* and *y* for the Bollinger Bands, respectively. Finally, loci *b*_*β*_*i*__^1^ through *b*_*β*_*i*__^*n*_*β*_*i*__^ represent the encoding of the weighting coefficient *β*_*i*_, *i* = 1, …, *m*.

In our encoding scheme, the chromosome representing the genotypes of parameters is to be transformed into the phenotype by (13) below for further fitness computation. The precision representing each parameter depends on the number of bits used to encode it in the chromosome, which is determined as follows:

(13) $$\begin{array}{c} y=\min_{y}+\frac{d}{2^{i}-1}\times \left(\max_{y}-\min_{y}\right), \end{array}$$

where *y* is the corresponding phenotype for the particular parameter; min⁡_*y*_ and max⁡_*y*_ are the minimum and maximum values of the parameter; *d* is the corresponding decimal value (*d* being truncated to integers if the parameter is of integer type), and *l* is the length of the block used to encode the parameter in the chromosome.

With this scheme, we define the fitness function of a chromosome as the annualized return of the trading system over *h* years of investment:

(14) $$\begin{array}{c} \text{fitness}=\sqrt[{h}]{R_{c}}, \end{array}$$

where *R*_*c*_ is the total cumulative return computed by (11).

**Figure 2:**
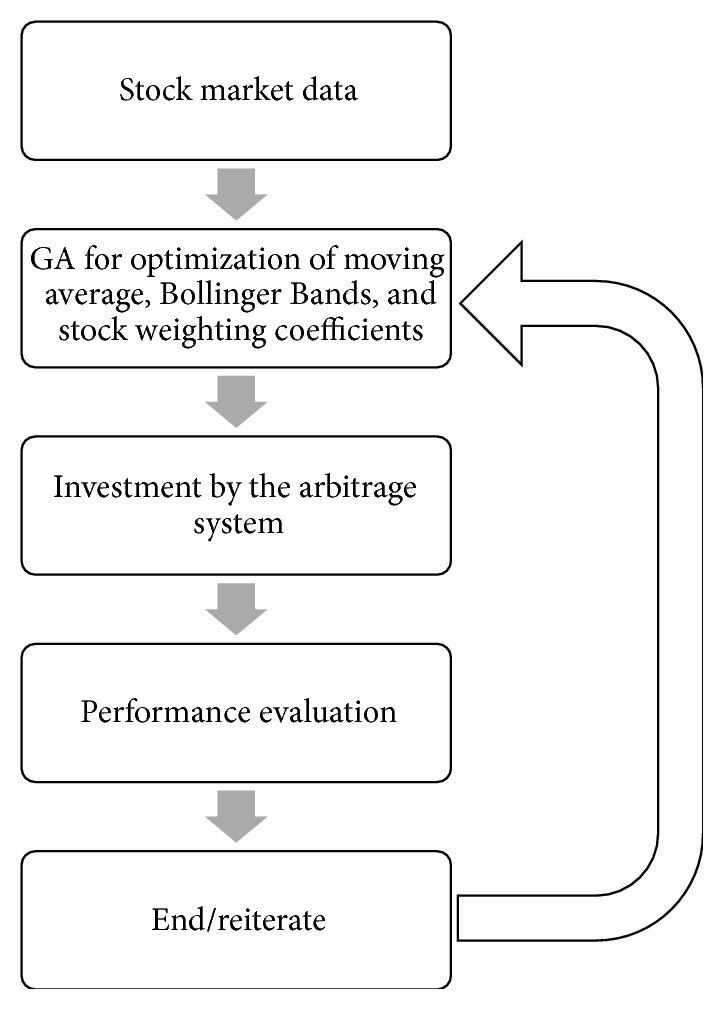
Flow chart of the GA-based arbitrage system.

Our overall GA-based arbitrage system is a multistage process, including the simultaneous optimization on the weighting coefficients for stocks, the period for the moving average, and the width of the Bollinger Bands. The input to the system is the time series datasets of stock price. For any given combinations of model parameters of the moving average, Bollinger Bands, and the weighting coefficients of stocks, we employ the pairs-trading arbitrage system for investment. In this work, the timing for trading is designated as buying (selling) the spread right after it gets to a certain distance (measured by standard deviations to the average) below (above) the average and the position is then closed right after the spread gets closer to the mean. The stocks to be long or short are determined by the weighting terms (*β*_*i*_'s) in the syntactic asset from (10). We then compute the corresponding returns for the performance evaluation of the system. In this study, the GA is used as the optimization tool for simultaneous optimization of these model parameters. The final output is a set of models parameters (optimized by the GA) that prescribes the pairs-trading and timing models. The flowchart of this GA-based trading system is summarized in Figure 2.

## 3. Results and Discussion

In this section we examine the performance of our proposed method for pair-trading systems. We use two sets of stocks listed in the Taiwan Stock Exchange for illustration: (1) the set of 10 stocks with similar characteristics from the semiconductor industry, which is the most important industrial sector in Taiwan over the past two decades, and (2) the set of the 10 stocks with largest market capitalization from various sectors, which denote distinct industrial characteristics in Taiwan.

### 3.1. 10 Stocks from the Semiconductor Industry

**Figure 3:**
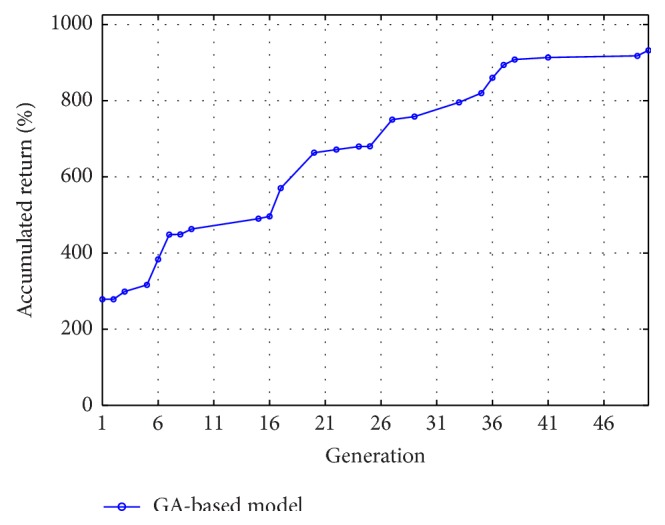
An illustration for the best-so-far curve by the GA.

The daily returns of the 10 semiconductor stocks in Taiwan from years 2003 to 2012 were used to examine the performance of the GA-optimized trading system.  Table 1 shows the 10 stocks used for this subsection.  Figure 3 displays an illustration of the best-so-far curve for the accumulated return (i.e., the total cumulative return) attained by the GA over 50 generations. (In order to study the quality of solutions over time, a traditional performance metric for the GA is the “best-so-far” curve that plots the fitness of the best individual that has been seen thus far by generation *n*, i.e., a point in the search space that optimizes the objective function thus far. In addition, in this study, the GA experiments employ a binary tournament selection [26], one-point crossover, and mutation rates of 0.7 and 0.005, resp. We also use 10 bits to encode each variable in the chromosome and use 50 individuals for the size of the population in each generation.) This figure shows how the GA searches for the solutions over the course of evolution to gradually improve the performance of the trading system.

**Figure 4:**
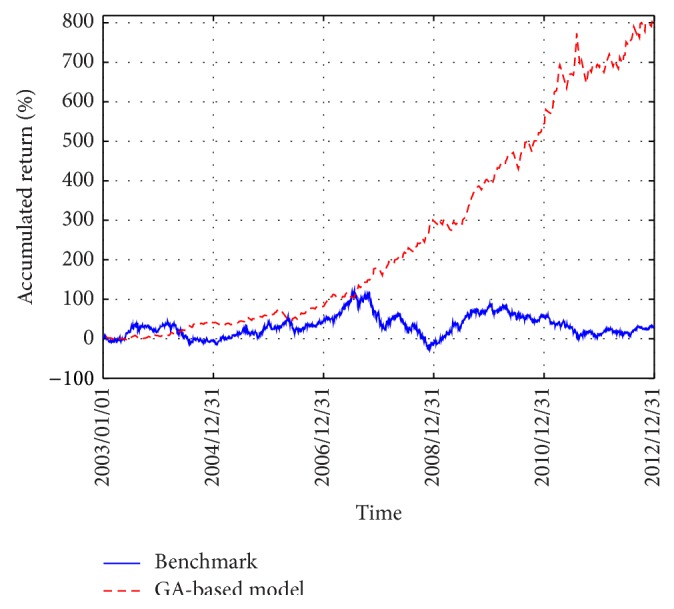
Accumulated return of the benchmark versus the GA-based model for the 10 semiconductor stocks from years 2003 to 2012.

Figure 4 displays an illustration of the accumulated return of the benchmark and that of our GA-based model. (In this study, the benchmark is defined as the traditional buy-and-hold method where we allocate one's capital in equal proportion to each stock and the accumulated return is calculated as the product of the average daily returns of all the 10 stocks over the 10 years; i.e., an investor invests all the capital in the stocks initially and sell all of them only at the end of the course of investment.) This figure shows that the GA-based model gradually outperforms the benchmark and the performance discrepancy becomes quite significant at the end of year 2012. As opposed to the buy-and-hold method that allocates one's capital in equal proportions to each stock, the GA proactively searches for the optimal proportions for long or short positions for each asset in order to construct the spread by (10). In addition, the GA also searches for the optimal timing for buying and shorting the stocks dynamically using the Bollinger Bands. In our study here, the weighting coefficients for the proportions of capital allocated to stocks, the period for the moving average, and the width of the Bollinger Bands are optimized simultaneously. As a result, in our proposed methodology, a trading system optimized by the GA is a composite of optimal arbitrage and market timing models. Thus, one may expect the GA to be advantageous to the construction of the arbitrage systems and Figure 4 indeed shows that the GA-based model outperforms the benchmark in the long run. Therefore, these results shed some light on how the optimization by the GA may be advantageous to the pairs-trading model.

**Figure 5:**
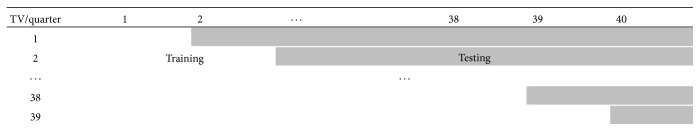
Temporal validation.

In order to further examine the validity of our proposed method, statistical validation on the models is conducted in this study. In reality, the learned model using the training data has to be tested by unseen data. Here, as shown in Figure 5, we use the stock data of the first several quarters to train the model, and the remaining data is used for testing. This setup is to provide a set of temporal validations to examine the effectiveness of the models in the dynamic environment of financial problems, which is different from the regular cross-validation procedure where the process of data being split into two independent sets is randomly repeated several times without taking into account the data's temporal order. However, in the financial study here, temporal order is critical since one would like to use all available data so far to train the model and to apply the models in the future for profits.

In the training phase of each TV, we conduct 50 runs for the GA and the best model learned from each run is examined in the testing phase. In both of the training and testing phases, the cumulative total return (accumulated return) of a model over the quarters is calculated and the corresponding annualized return is computed by (14). The annualized returns of the best 50 models in each TV are then averaged and displayed for the training and testing phases in Table 2. In this table, we also provide the annualized benchmark return for further comparison with the GA-based models, where the cumulative total return for the benchmark is calculated from the product of the average quarterly returns of the 10 semiconductor stocks over the period of time in training or testing, and the corresponding annualized return is again computed by (14).

**Figure 6:**
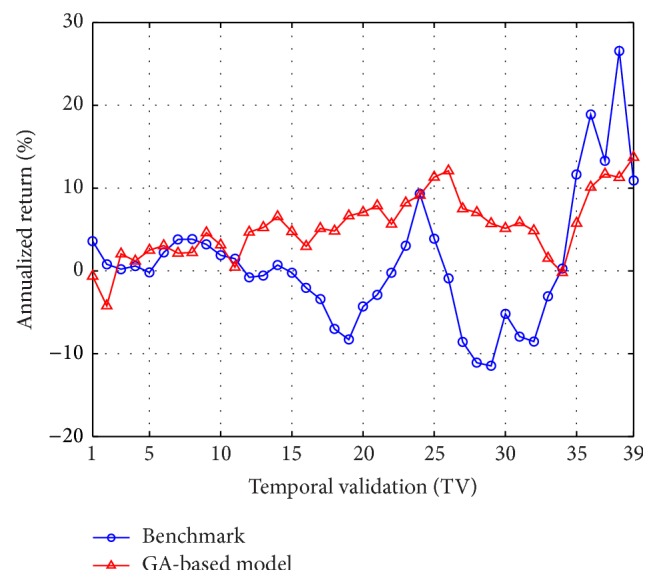
Averaged annualized return of the top 50 GA-based models versus the benchmark (in each TV of the testing phase) for the 10 semiconductor stocks from years 2003 to 2012.

In Table 2, an inspection on the means of annualized model returns shows that in all the 39 TVs of the training case the GA-based method outperforms the benchmark. For the testing phase, in 30 out of 39 cases the GA-based method outperforms the benchmark.  Figure 6 further displays a visual gist on this performance discrepancy of the two methods in the testing phase. As can be seen, in most of the TVs, the annualized return of the GA-based model is larger than that of the benchmark. These results thus demonstrate our GA-based method is promising for solving the pairs-trading problem.

### 3.2. 10 Stocks with the Largest Market Capitalization

Next we use the 10 stocks of the largest market capitalization listed in the Taiwan Stock Exchange to further examine our proposed method. The daily returns of stocks from years 2003 to 2012 were again used for the optimization task by the GA.  Table 3 shows the 10 stocks with the largest market cap used in this study.

**Figure 7:**
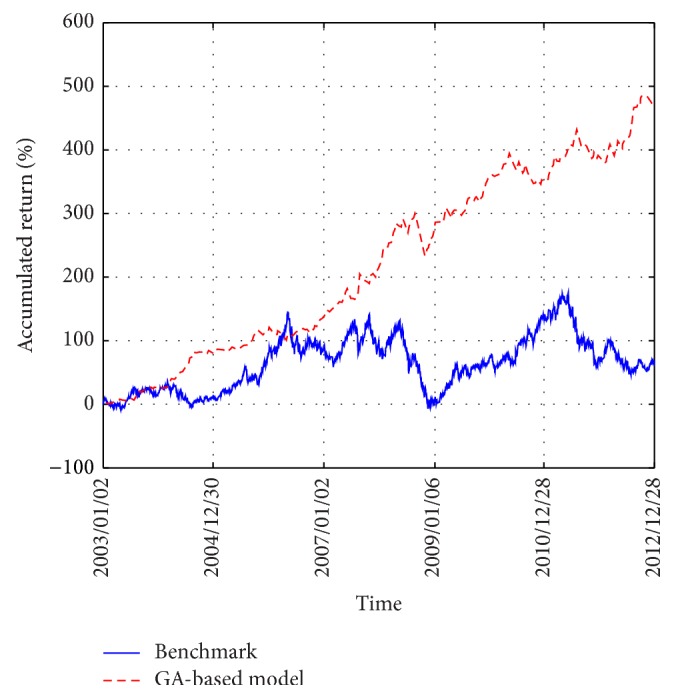
Accumulated return of the benchmark versus the GA-based model for the 10 largest market cap stocks from years 2003 to 2012.

Figure 7 displays an illustration of the accumulated return of the benchmark (which is again defined as the product of the average daily returns of the 10 largest market cap stocks over the 10 years) and that of our GA-based model. As can be seen, the GA-based model gradually outperforms the benchmark over the course of investment during years 2003 to 2012, and the performance discrepancy becomes significant at the end of year 2012. This figure thus illustrates how the GA-based model may outperform the benchmark in the long run.

**Figure 8:**
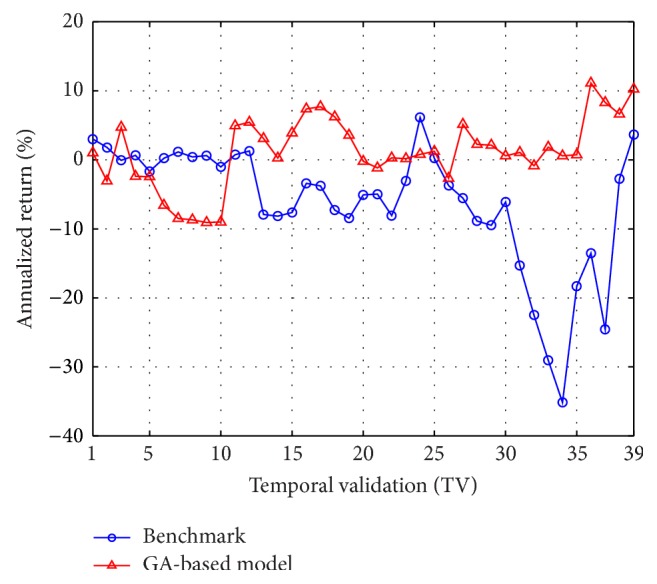
Averaged annualized return of the top 50 GA-based models versus the benchmark (in each TV of the testing phase) for the 10 largest market cap stocks from years 2003 to 2012.

For the temporal validation, by the same procedure used in the previous subsection, Table 4 shows the annualized benchmark return and the average of the annualized model returns for the training and testing cases. As can be seen from the means of the annualized model returns in the training case, the GA-based method outperforms the benchmark in all the 39 TVs. For the testing phase, in 29 out of 39 cases the GA-based method outperforms the benchmark, as well.  Figure 8 then displays the results in Table 4 for each TV in the testing phase. An inspection of Figure 8 thus shows that, in 29 out of 39 TVs, the GA-based models outperform the benchmark in terms of annualized returns.

### 3.3. Model Robustness

Finally, we examine the robustness of the models generated by our method using the measure of precision studied in [5], which is defined as

(15) $$\begin{array}{c} \text{Precision}=\frac{\text{TP}}{\text{TP}+\text{FP}}. \end{array}$$

In this definition, TP and FP denote the number of true positives and false positives, respectively. In this study, a true positive occurs when a model outperforms the benchmark in training, and it later turns out to outperform the benchmark in testing, as well; otherwise, the model generates a false positive. This statistic is an important metric that indicates whether our proposed method can generate robust models when the problem is in a dynamic environment, such as the financial problem studied here.

Typically, if a method generates a model that outperforms the benchmark in the training phase, one would like the model to continue to outperform the benchmark in the testing phase. Therefore, if our proposed method is able to generate many true positives that leads to high precision, it is an indication that our method is effective in generating robust models.  Table 5 displays the results of precision for the 10 semiconductor and largest market cap stocks. As can be seen, the results show that the precision of our proposed method is more than 0.7 in both cases, thereby indicating that our proposed method is indeed effective.

## 4. Conclusions

In this paper, we presented a GA-based methodology for the application of pairs trading in computational finance. In order to examine the validity of the proposed methodology, we conducted a statistical validation on the learned models to account for the temporal order and dynamic characteristics of the stock data, which is critical for the real-world investment as practically one expects the models constructed to gain profits in the future. Through the optimization of parameters of the trading models for a group of stocks, the experimental results showed that our GA-based method is able to significantly outperform the benchmark and can generate robust models for pairs trading. We thus expect this GA-based method to advance the research in computational intelligence for financial applications and provide a promising solution to pairs trading.

## Figures and Tables

**Table 1:** The 10 semiconductor stocks used in this study.

Ticker	Name (Chinese)	Name (English)
2325	矽品	Siliconware Precision Industries Co, Ltd.
2330	臺積電	Taiwan Semiconductor Manufacturing
2303	聯電	United Microelectronics Corp.
2311	日月光	Advanced Semiconductor Engineering, Inc.
2337	旺宏	Macronix International Company, Ltd.
2301	光寶科	Lite-On Technology Corp.
2308	台達電	Delta Electronics, Inc.
2409	友達	AU Optronics Corporation
2451	創見	Transcend Information, Inc.
2454	聯發科	MediaTek Inc.

**Table 2:** Comparisons of the annualized returns of the GA-based model and the benchmark for the 10 semiconductor stocks from years 2003 to 2012.

TV	Training period	Annualized benchmark return	Mean of annualized model return	Standard deviation	Testing period	Annualized benchmark return	Mean of annualized model return	Standard deviation
1	2003Q1	−27.23%	157.55%	0.0494	2003Q2–2012Q4	3.60%	0.01%	0.5125
2	2003Q1–2003Q2	36.06%	137.35%	0.1117	2003Q3–2012Q4	0.80%	−4.42%	0.5494
3	2003Q1–2003Q3	39.79%	119.60%	0.1119	2003Q4–2012Q4	0.21%	3.49%	0.5392
4	2003Q1–2003Q4	20.67%	85.98%	0.1498	2004Q1–2012Q4	0.59%	2.05%	0.4707
5	2003Q1–2004Q1	24.25%	72.06%	0.1553	2004Q2–2012Q4	−0.19%	2.55%	0.4833
6	2003Q1–2004Q2	5.26%	59.23%	0.1626	2004Q3–2012Q4	2.22%	5.14%	0.7364
7	2003Q1–2004Q3	−2.64%	55.70%	0.3206	2004Q4–2012Q4	3.80%	4.49%	0.6824
8	2003Q1–2004Q4	−1.33%	53.64%	0.3889	2005Q1–2012Q4	3.83%	5.17%	0.8286
9	2003Q1–2005Q1	0.03%	45.19%	0.3904	2005Q2–2012Q4	3.20%	2.58%	0.6446
10	2003Q1–2005Q2	4.49%	42.85%	0.4555	2005Q3–2012Q4	1.88%	3.24%	0.6986
11	2003Q1–2005Q3	5.36%	42.02%	0.4171	2005Q4–2012Q4	1.48%	1.58%	0.4788
12	2003Q1–2005Q4	11.17%	34.85%	0.3509	2006Q1–2012Q4	−0.77%	4.70%	0.5345
13	2003Q1–2006Q1	9.63%	34.61%	0.2917	2006Q2–2012Q4	−0.57%	5.29%	0.5652
14	2003Q1–2006Q2	6.65%	32.14%	0.2780	2006Q3–2012Q4	0.71%	5.92%	0.5332
15	2003Q1–2006Q3	7.54%	31.70%	0.3518	2006Q4–2012Q4	−0.22%	4.58%	0.4451
16	2003Q1–2006Q4	9.38%	30.56%	0.3527	2007Q1–2012Q4	−2.03%	4.16%	0.3340
17	2003Q1–2007Q1	11.74%	30.21%	0.4362	2007Q2–2012Q4	−3.41%	5.25%	0.3628
18	2003Q1–2007Q2	15.86%	27.98%	0.5660	2007Q3–2012Q4	−7.02%	8.11%	0.6571
19	2003Q1–2007Q3	16.65%	31.03%	0.7558	2007Q4–2012Q4	−8.30%	7.48%	0.4423
20	2003Q1–2007Q4	10.80%	32.33%	0.7995	2008Q1–2012Q4	−4.31%	7.86%	0.5680
21	2003Q1–2008Q1	7.98%	31.33%	1.0381	2008Q2–2012Q4	−2.88%	8.24%	0.3712
22	2003Q1–2008Q2	5.50%	35.78%	1.4992	2008Q3–2012Q4	−0.23%	7.87%	0.3083
23	2003Q1–2008Q3	2.35%	34.33%	1.9692	2008Q4–2012Q4	3.05%	6.84%	0.2409
24	2003Q1–2008Q4	−2.29%	34.66%	1.9921	2009Q1–2012Q4	9.29%	8.63%	0.2430
25	2003Q1–2009Q1	1.88%	31.07%	1.7270	2009Q2–2012Q4	3.90%	9.78%	0.2099
26	2003Q1–2009Q2	4.43%	29.88%	1.6957	2009Q3–2012Q4	−0.90%	10.43%	0.2683
27	2003Q1–2009Q3	8.62%	31.06%	2.1604	2009Q4–2012Q4	−8.58%	8.68%	0.2157
28	2003Q1–2009Q4	9.09%	31.06%	2.3144	2010Q1–2012Q4	−11.09%	6.66%	0.1684
29	2003Q1–2010Q1	8.39%	31.53%	2.3984	2010Q2–2012Q4	−11.47%	5.74%	0.2323
30	2003Q1–2010Q2	5.83%	30.06%	2.5543	2010Q3–2012Q4	−5.19%	5.89%	0.1809
31	2003Q1–2010Q3	5.83%	27.71%	2.2193	2010Q4–2012Q4	−7.93%	6.05%	0.1847
32	2003Q1–2010Q4	5.80%	27.26%	2.7974	2011Q1–2012Q4	−8.53%	5.48%	0.1443
33	2003Q1–2011Q1	3.86%	29.38%	2.8889	2011Q2–2012Q4	−3.09%	1.32%	0.1056
34	2003Q1–2011Q2	3.11%	28.69%	3.3213	2011Q3–2012Q4	0.29%	0.20%	0.1043
35	2003Q1–2011Q3	1.82%	27.14%	2.7034	2011Q4–2012Q4	11.66%	5.24%	0.0842
36	2003Q1–2011Q4	1.22%	27.73%	3.7890	2003Q1–2012Q4	18.89%	11.33%	0.0745
37	2003Q1–2012Q1	2.03%	25.98%	3.3508	2003Q1–2012Q4	13.27%	12.24%	0.0604
38	2003Q1–2012Q2	1.58%	25.08%	3.2671	2003Q1–2012Q4	26.57%	11.28%	0.0582
39	2003Q1–2012Q3	2.50%	25.38%	3.6955	2012Q4	10.91%	12.68%	0.0414

**Table 3:** The 10 largest market cap stocks used in this study.

Ticker	Name (Chinese)	Name (English)
2330	臺積電	Taiwan Semiconductor Manufacturing
2317	鴻海	Hon Hai Precision
2454	聯發科	MediaTek Inc.
1301	臺塑	Formosa Plastics
1303	南亞	Nan Ya Plastics
1326	台化	Formosa Chemicals
2412	中華電	Chunghwa Telecom
2882	國泰金	Cathay Financial Holding
2308	台達電	Delta Electronics, Inc.
2008	高興昌	Kao Hsing Chang Iron

**Table 4:** Comparisons of the annualized returns of the GA-based model and the benchmark for the 10 largest market cap stocks from years 2003 to 2012.

TV	Training period	Annualized benchmark return	Mean of annualized model return	Standard deviation	Testing period	Annualized benchmark return	Mean of annualized model return	Standard deviation
1	2003Q1	−9.13%	133.86%	0.3965	2003Q2–2012Q4	33.58%	1.03%	0.0472
2	2003Q1–2003Q2	18.81%	109.67%	0.1066	2003Q3–2012Q4	18.51%	−3.06%	0.0348
3	2003Q1–2003Q3	33.87%	86.87%	0.1665	2003Q4–2012Q4	−0.69%	4.74%	0.0571
4	2003Q1–2003Q4	26.40%	54.91%	0.0853	2004Q1–2012Q4	6.10%	−2.38%	0.0344
5	2003Q1–2004Q1	31.57%	52.05%	0.0939	2004Q2–2012Q4	−13.55%	−2.43%	0.0558
6	2003Q1–2004Q2	16.71%	60.24%	0.1204	2004Q3–2012Q4	1.99%	−6.57%	0.0407
7	2003Q1–2004Q3	11.80%	62.13%	0.0971	2004Q4–2012Q4	9.94%	−8.50%	0.0273
8	2003Q1–2004Q4	15.29%	55.99%	0.1029	2005Q1–2012Q4	3.50%	−8.68%	0.0305
9	2003Q1–2005Q1	12.64%	49.95%	0.0850	2005Q2–2012Q4	4.91%	−9.07%	0.0449
10	2003Q1–2005Q2	15.71%	40.65%	0.0636	2005Q3–2012Q4	−7.39%	−9.01%	0.0498
11	2003Q1–2005Q3	6.79%	37.80%	0.0596	2005Q4–2012Q4	5.58%	4.94%	0.0401
12	2003Q1–2005Q4	12.54%	35.57%	0.0478	2006Q1–2012Q4	9.46%	5.46%	0.0724
13	2003Q1–2006Q1	29.21%	30.42%	0.0591	2006Q2–2012Q4	−42.67%	3.09%	0.0614
14	2003Q1–2006Q2	32.17%	39.09%	0.0536	2006Q3–2012Q4	−42.45%	0.26%	0.0623
15	2003Q1–2006Q3	23.30%	32.03%	0.0724	2006Q4–2012Q4	−39.08%	3.91%	0.0655
16	2003Q1–2006Q4	9.71%	28.78%	0.0284	2007Q1–2012Q4	−18.77%	7.38%	0.0341
17	2003Q1–2007Q1	10.42%	28.97%	0.0271	2007Q2–2012Q4	−19.82%	7.71%	0.0382
18	2003Q1–2007Q2	15.69%	27.25%	0.0357	2007Q3–2012Q4	−33.97%	6.24%	0.0324
19	2003Q1–2007Q3	16.14%	29.75%	0.0323	2007Q4–2012Q4	−37.09%	3.59%	0.0397
20	2003Q1–2007Q4	11.22%	31.29%	0.0383	2008Q1–2012Q4	−22.89%	−0.22%	0.0292
21	2003Q1–2008Q1	9.96%	33.59%	0.0436	2008Q2–2012Q4	−21.45%	−1.16%	0.0466
22	2003Q1–2008Q2	13.56%	28.81%	0.0756	2008Q3–2012Q4	−31.51%	0.29%	0.0438
23	2003Q1–2008Q3	8.29%	27.84%	0.0506	2008Q4–2012Q4	−12.44%	0.18%	0.0357
24	2003Q1–2008Q4	1.30%	24.99%	0.0535	2009Q1–2012Q4	26.95%	0.81%	0.0501
25	2003Q1–2009Q1	5.04%	23.92%	0.0516	2009Q2–2012Q4	0.86%	1.23%	0.0725
26	2003Q1–2009Q2	7.37%	25.11%	0.0395	2009Q3–2012Q4	−12.45%	−2.67%	0.0802
27	2003Q1–2009Q3	6.73%	24.79%	0.0252	2009Q4–2012Q4	−16.92%	5.16%	0.0357
28	2003Q1–2009Q4	7.63%	25.15%	0.0215	2010Q1–2012Q4	−24.31%	2.24%	0.0326
29	2003Q1–2010Q1	7.14%	25.32%	0.0201	2010Q2–2012Q4	−23.97%	2.14%	0.0378
30	2003Q1–2010Q2	7.28%	23.47%	0.0337	2010Q3–2012Q4	−14.58%	0.59%	0.0753
31	2003Q1–2010Q3	9.81%	21.78%	0.0373	2010Q4–2012Q4	−31.17%	1.08%	0.0947
32	2003Q1–2010Q4	11.29%	20.65%	0.0395	2011Q1–2012Q4	−39.94%	−0.85%	0.0970
33	2003Q1–2011Q1	12.29%	21.09%	0.0343	2011Q2–2012Q4	−45.11%	1.84%	0.0532
34	2003Q1–2011Q2	13.44%	20.05%	0.0388	2011Q3–2012Q4	−47.78%	0.58%	0.0600
35	2003Q1–2011Q3	7.56%	21.41%	0.0359	2011Q4–2012Q4	−22.33%	0.75%	0.1054
36	2003Q1–2011Q4	8.08%	18.04%	0.0389	2003Q1–2012Q4	−13.53%	11.13%	0.0909
37	2003Q1–2012Q1	6.51%	19.60%	0.0388	2003Q1–2012Q4	−19.06%	8.32%	0.0801
38	2003Q1–2012Q2	4.77%	19.41%	0.0253	2003Q1–2012Q4	−1.38%	6.64%	0.1144
39	2003Q1–2012Q3	3.43%	19.58%	0.0171	2012Q4	0.91%	10.26%	0.1145

**Table 5:** Precision for the 10 semiconductor and largest market cap stocks.

	10 semiconductor stocks	10 largest market cap stocks
Precision	0.7692	0.7180
